# External Validation of the New 2023 International Federation of Gynecology and Obstetrics Staging System in Endometrial Cancer Patients: 12-Year Experience from an European Society of Gynecological Oncology-Accredited Center

**DOI:** 10.3390/medicina60091421

**Published:** 2024-08-30

**Authors:** Dimitrios Tsolakidis, Dimitrios Zouzoulas, Iliana Sofianou, Tilemaxos Karalis, Kimon Chatzistamatiou, Vasilis Theodoulidis, Maria Topalidou, Eleni Timotheadou, Grigoris Grimbizis

**Affiliations:** 1 1st Department of Obstetrics & Gynecology, Aristotle University of Thessaloniki, “Papageorgiou” Hospital, 564 29 Thessaloniki, Greece; 2 Radiotherapy Department, “Papageorgiou” Hospital, 564 29 Thessaloniki, Greece; 3 Department of Oncology, Aristotle University of Thessaloniki, “Papageorgiou” Hospital, 564 29 Thessaloniki, Greece

**Keywords:** endometrial cancer, cancer staging, prognosis, survival

## Abstract

*Background and Objectives*: The new molecular classification of endometrial cancer continuously changes the management of the disease in everyday clinical practice. Recently, FIGO released a new staging system for endometrial cancer, which incorporates molecular substages and subdivides further early-stage disease. The aim of this study was to investigate the differences between the two FIGO staging systems and evaluate the prognostic precision of the new one. *Materials and Methods*: We retrospectively analyzed the records of patients with endometrial cancer that were fully treated in the 1st Department of Obstetrics & Gynecology, in 2012–2023. Patient characteristics, oncological outcome, and follow-up information were collected. The primary outcomes were the stage shifts and the survival data. *Results*: Sixty-seven (15.5%) patients had a stage shift and the majority of them concerned early-stage disease and specifically an upshift from 2009 stages IA and IB to 2023 stage IIC. Concerning survival, a better median and 5-year PFS was present in stage II disease, and when comparing the prognostic precision of the two FIGO staging systems no significant difference was present. *Conclusions*: The new 2023 FIGO staging system better distinguishes early-stage endometrial cancer into its prognostic groups and seems to be as precise as the old 2009 FIGO staging system.

## 1. Introduction

Endometrial cancer is the fourth most common cancer in women in the United States. The estimated number of new endometrial carcinoma cases in women in the United States in 2023 was 66,200 (7%) with 13,030 (5%) deaths. The incidence rate has been rising with aging and changes in lifestyle due to an increase percentage of obesity in the women’s population [1]. Histologically, endometrial carcinoma begins from the lining of the uterus. It has been broadly classified into two clinicopathogenetic subgroups in 1983 by Bokhman [2]. Type I, including endometrioid tumors, which represent the majority of uterine tumors and are estrogen-driven with lower-grade, less myometrial invasion, and a more favorable prognosis compared with type II, which are histologically and clinically more advanced with a higher grade and poor prognosis. Although, the majority of women with endometrial carcinoma have good prognosis due to its detection in early stages, the disease-free survival rate of high-grade tumors may be as low as 13% after adjuvant chemotherapy [3]. Thus, it is important to identify prognostic factors for the adequate therapy of endometrial carcinoma.

The clinicopathological prognostic factors have low reproducibility between expert pathologists, even with the use of immunohistochemistry. In 2020, the new WHO classification tried to avoid discrepancies by using morphological, immunohistochemical, and molecular diagnostic features [4]. This situation resulted in over-treatment and under-treatment of thousands of cases among cancer centers globally [5,6,7]. In 2013, The Cancer Genome Atlas (TCGA) Research Network settled on a molecular classification of endometrial cancer to anticipate the prognosis and correct therapy [8]. The four genomic subgroups, based on a combination of mutation and protein expression analyses, were the DNA polymerase epsilon (POLE) mutated subtype, the mismatch repair-deficient subtype (MMRd, MSI), no specific molecular profile (NSMP), and p53 abnormal (p53 mutation type) [9]. These four subgroups were combined to create this new molecular classification, together with the old classification, in the new ESGO/ESTRO/ESP guidelines for the exceptional management of endometrial cancer patients [10].

All these data empower the prognostic significance of molecular classification with clinicopathological factors to change the International Federation of Gynecology and Obstetrics (FIGO) staging system for endometrial cancer. The new 2023 FIGO staging system [11] of endometrial cancer is more complicated than that of 2009 [12]. Although, the cornerstone of the staging of any cancer is the anatomic involvement, new elements are integrated in the new 2023 FIGO, such as histologic grade, histology, lymphovascular space invasion (LVSI), and molecular classification. Thus, this produces a noticeable divergence in the staging of FIGO 2009. The aim of this study was to investigate the stage shifts between the two staging systems and to evaluate the clinical and prognostic impact of the new 2023 FIGO staging system.

## 2. Materials and Methods

### 2.1. Study Characteristics

We retrospectively reviewed the medical records of all women with newly diagnosed endometrial cancer, who were treated in the 1st Department of Obstetrics & Gynecology, AUTh, “Papageorgiou” General Hospital, from 1 January 2012 until 31 December 2023 and identified those that received their complete treatment at our hospital. In total, 476 patients were diagnosed with endometrial cancer during this period of time. A written approval was received from the Institutional Review Board of the hospital.

### 2.2. Patients

Inclusion criteria:

- Histological confirmation of endometrial cancer.
- Complete treatment at our hospital.

Exclusion criteria:

- Synchronous neoplasm.
- Recurrent endometrial cancer.
- Missing important registry data.

As a result of the above-mentioned criteria, 30 out of the 476 women with endometrial cancer were excluded due to synchronous neoplasms or as a recurrence of endometrial cancer. Moreover, 16 women were excluded because they were missing important registry data and could not be further statistically analyzed. Hence, finally, 431 women with endometrial cancer were identified as eligible for further analysis, with no duplicate data and important missing values.

All patients underwent a complete laboratory and imaging staging for endometrial cancer and after an MDT board meeting were operated based on the presumed stage of the disease. In early stages, minimal invasive surgery was performed in the majority of the cases, while some underwent laparotomy. Total hysterectomy with or without bilateral salpingo-oophecetomy and omentectomy, in specific histological types, were offered. Lymph node staging was performed with a sentinel lymph node biopsy and in some cases, with pelvic and/or paraaortic lymph node dissection. In advanced stages, a cytoreductive surgery was performed. Adjuvant treatment was decided after the MDT board meeting according to international guidelines [10]. A close follow-up of the patients included a clinical examination and laboratory and imaging exams.

### 2.3. Data Collection

Data were collected during a period of one month. Our Gynecological–Oncology Unit has an online registry with all the relevant data of the patient’s medical records. In order to avoid inconsistencies among different dates of data collection, a uniform data collection sheet (excel file) was used during the retrospective mining of the patient’s medical records. The data sheet included the following information:

- Patient’s identifiers:
  ○ Name.
  ○ Hospital identification number.
- Patient’s age.
- Body Mass Index (BMI).
- Charlson Comorbidity Index (CCI).
- Histological type.
- Lymphovascular space invasion (LVSI).
- Tumor grade.
- FIGO staging (2009).
- FIGO staging (2023).
- Molecular classification:
  ○ DNA polymerase epsilon (POLE) mutation.
  ○ Mismatch repair-deficient subtype or microsatellite instability (MMRd or MSI).
  ○ p53 abnormal (mutation type).
- Time-related data:
  ○ Date of diagnosis.
  ○ Date of recurrence.
  ○ Date of last follow-up or death.

LVSI was defined as no, focal, and substantial according to the latest WHO classification. Focal was defined as the presence of a single focus around the tumor and substantial as a multifocal or diffuse arrangement or the presence of tumor cells in five or more lymphovascular spaces [13]. Furthermore, tumor grade was categorized with a binary system as low or high [14], and all patients were classified firstly by the 2009 FIGO staging system [12] and then by the 2023 one [15]. The two FIGO staging systems are presented in Table 1 and Table 2. All stage shifts were documented: upstaging was considered a reclassification to a higher group and downstaging to a lower group.

Molecular testing for the integration of the new classification was performed. POLE sequencing with NGS was performed to test 11 POLE exonuclease domain hotspots for mutations: DNA was isolated from the sample under examination, and a mutational analysis of the region of the POLE gene (exons 9–14) was performed. Sequencing was conducted using the Ion Gene Studio S5 Prime System Next Generation Sequencing platform (Thermo Fisher Scientific, Waltham, MA, USA). An MSI assay to test for microsatellite instability was conducted: Genomic DNA was isolated from the paraffin-embedded tumor tissue sample. This was followed by an analysis of 76 markers by next-generation sequencing to assess the microsatellite instability (MSI) status using Ion Ampliseq technology. Sequencing was performed using the Ion Gene S5 Prime System next-generation sequencing platform (Thermo Fisher Scientific). The test provides results for individual microsatellites and produces an MSI score for the sample. A sample is considered positive for MSI if the microsatellite instability score is greater than 30. p53 immunohistochemistry testing to identify the 4 distinct mutant-expression patterns was conducted: There are four distinct p53 mutant-expression patterns: diffuse and strong nuclear positivity, complete absence of expression, overexpression in the cytoplasm, and a well-delimited area of the tumor with mutant expression of p53 in a background of wild-type expression. In cases of multiple classifiers, meaning the presence of more than one molecular feature, the more favorable prognostic group was retained [16].

### 2.4. Statistical Analysis

All analyses were performed using RStudio, version 4.3.0. For descriptive statistics of qualitative variables, the frequency distribution procedure was run with a calculation of the number of cases and percentages. On the other hand, for descriptive statistics of quantitative variables, the mean, median, range, and standard deviation were used to describe central tendency and dispersion. Progression-free (DFS) and overall survival (OS) analyses were performed using the Kaplan—Meier curves. The 5-year PFS and OS survival were assessed per cancer stage between the 2009 and 2023 FIGO staging systems. Progression-free survival was defined as the time interval between the date of diagnosis and the date of first recurrence or disease progression, while overall survival was defined as the time interval from diagnosis to the date of death or last follow-up. To evaluate the prognostic precision of each FIGO staging system, Cox proportional hazard models between the 2 FIGO staging systems were used. The Akaike information criterion (AIC), the Bayesian information criterion (BIC), and Harrel’s concordance index (C-index) were calculated for each model. The likelihood ratio test was performed in order to test whether using the 2023 FIGO staging system as a predictor provided a better model fit. A *p*-value of <0.05 was considered as statistically significant.

## 3. Results

This retrospective cohort study included 476 women, who were treated during the period of the study for endometrial cancer in the Gynecological–Oncology Unit, 1st Department of Obstetrics & Gynecology, Aristotle University of Thessaloniki, “Papageorgiou” General Hospital. After screening the patients based on the inclusion and exclusion criteria, 431 patients were eligible for further analysis in this study.

Patients’ characteristics are outlined in Table 3. The mean age of the women at the time of diagnosis was 63.5 years old, while the mean BMI was 32.8 kg/m^2^, meaning that most women were obese. Furthermore, concerning the performance status of our patients, almost half of them (47%) had moderate comorbidities, which was measured by the Charlson Comorbidities Index (CCI). These results are in accordance with the phenotype of the disease. Concerning histology, the majority of the patients had low-grade endometrioid neoplasms with no lymphovascular space invasion (LVSI). Unfortunately, only a fraction of our patients (7.7%) were fully tested for the new molecular classification.

**Figure 1:**
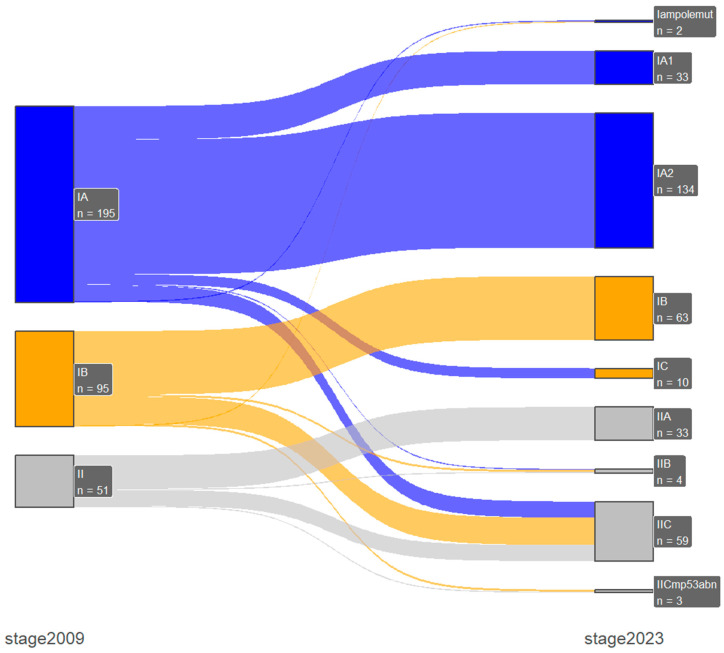
Early-stage disease Shankey plot for stage shifts between 2009 and 2023 FIGO staging systems.

**Figure 2:**
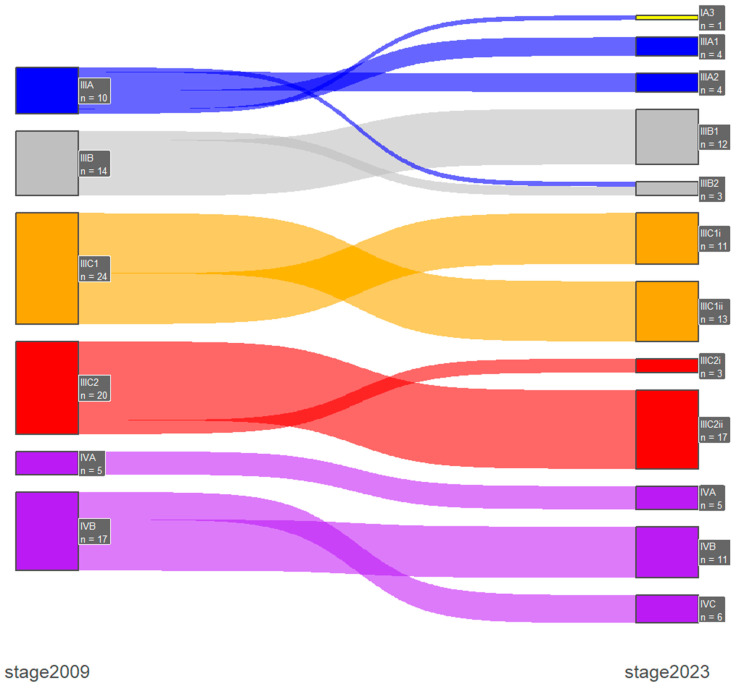
Advanced-stage disease Shankey plot for stage shifts between 2009 and 2023 FIGO staging systems.

Stage shifts between the two FIGO staging systems, which was one of the primary endpoints of our study, was present in 67 patients (15.5%). Specifically, there were 2 (0.5%) downshifts and 65 (15%) upshifts, with most of them occurring only between early-stage or advanced-stage disease and only one case of downshifts from advanced- to early-stage disease. In early-stage disease (FIGO stage I/II), the majority of the upshifts were from 2009 FIGO stage IA (n = 16) and IB (n = 27) to the new 2023 FIGO stage IIC substage, and with the incorporation of the molecular profile, there were three stage shifts from the 2009 FIGO stage IB: one downshift to IAm_polemut_ and two upshifts to IICm_p53abn_. Furthermore, in advanced-stage disease (FIGO stage III/IV), the majority of the upshifts were from 2009 FIGO stage IVB (n = 6) to 2023 FIGO stage IVC. These results are presented in Table 4 and in Figure 1 and Figure 2.

**Figure 3:**
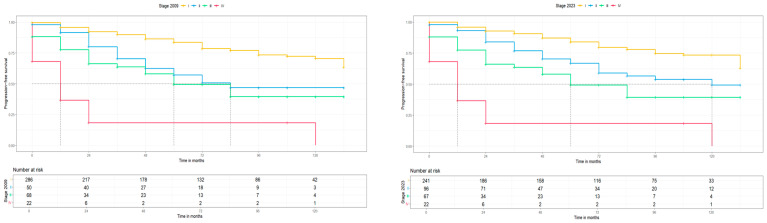
PFS for 2009 and 2023 FIGO staging systems (no molecular classification).

**Figure 4:**
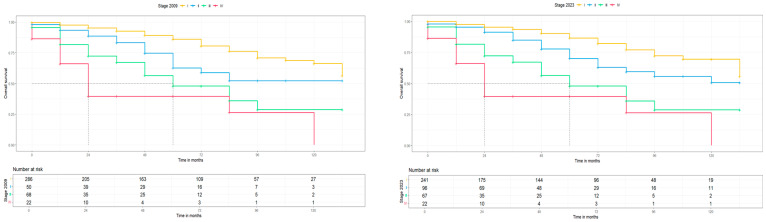
OS for 2009 and 2023 FIGO staging systems (no molecular classification).

Moreover, the second primary endpoint of this study was the clinical and prognostic impact of the new 2023 FIGO staging system. For this reason, the 5-year progression free survival (PFS) and the 5-year overall survival (OS) for the 2009 and 2023 FIGO staging systems were calculated and Kaplan–Meier curves for PFS and OS were constructed, respectively. The median follow-up of this cohort was 48 months with an IQR of 12–72, which is long enough to provide reliable survival results. For stage I disease, the 5-year PFS and 5-year OS were slightly better for the 2023 FIGO staging system (83.3 (78.8, 88.4) to 84 (78.9, 89.5) and 86 (81.2, 91.2) to 86.6 (81.5, 92.1), with an interval rate change of +0.7% and +0.6%, respectively) compared to the 2009 FIGO staging system, but the median PFS and OS for both staging systems were >145 months. For stage II disease, a notably higher 5-year PFS and 5-year OS was found for the 2023 FIGO staging system [57.2 (43.8, 74.6) to 66.9 (56.7, 79.0) and 62.8 (48.9, 80.7) to 70.2 (59.6, 82.8), with an interval rate change of +9.7% and +7.4%, respectively] compared to the 2009 FIGO staging system. Concerning survival rates, a significantly higher median PFS (*p* < 0.05) was observed in the 2023 FIGO staging system (2009: 84 vs. 2023: 120 months), but with no differences in OS (>145 months for both). For advanced-stage disease, similar 5-year PFS and 5-year OS were found between the two FIGO staging systems (for substages of stage III and stage IV, no statistical analysis was possible due to the low case numbers) and no difference in the median PFS and OS, respectively. These results are summarized in Table 5 and Figure 3 and Figure 4.

In addition, we further analyzed the group of patients with the majority of stage shifts between the two staging systems. Specifically, 48 patients were upshifted from 2009 FIGO stage I to 2023 FIGO stage II. The main reason for these upshifts was substage IIC, which includes all aggressive histological subtypes with any myometrial invasion: high-grade endometrioid, serous, clear cell, undifferentiated, mixed, mesonephric-like, gastrointestinal mucinous-type carcinomas, and carcinosarcomas. Based on the histopathological features, half (n = 24, 50%) of this group of patients had endometrioid tumors, while only seven (14.6%) patients had low-grade tumors, and only five (10.4%) substantial LVSI. The aforementioned data are presented in Table 6. Looking into the survival rates of this group of patients: 5-year DFS was 76.4 (62, 94) and 5-year OS was 81.9 (68.3, 98.2) months.

The AIC, BIC, C-index, and likelihood-ratio test were all used to compare the prognostic precision of the two FIGO staging systems. The AIC scores to predict PFS for the 2023 FIGO staging system compared to the 2009 FIGO staging system were 1260.42 and 1256.31, respectively. Furthermore, the BIC scores to predict PFS for the 2023 and 2009 FIGO staging systems were 1274.35 and 1264.67, respectively. The C-index for both FIGO staging systems were 0.71 and 0.70, respectively, for PFS. The likelihood ratio comparing the two models showed that there was no statistically significant difference (*p* = 0.95). On the other hand, for the OS between the 2023 FIGO staging system and the 2009 FIGO staging system, the AIC scores were 1047.26 and 1043.5, respectively, the BIC scores were 1060.44 and 1051.48, respectively, and the C-index was 0.71 for each model. The likelihood-ratio test comparing the two models did not show any significant difference either (*p* = 0.86).

## 4. Discussion

The primary objective of our study was to investigate the stage shifts between the new 2023 FIGO staging system compared to the old 2009 one and to assess its clinical and prognostic impact. We designed a retrospective study of endometrial cancer patients that were accurately staged with the 2009 FIGO staging system and re-staged them according to the new 2023 system, including the molecular classification when it was available.

This study included 431 patients with endometrial cancer, which were all included in the final analyses, but full molecular testing and therefore the implementation of the new molecular classification was only possible in a fraction of our cohort (7.7%). Our cohort had a long follow-up period with a median of 48 months, and no important histological data were missing from any patient. Stage shifts were present in 15.5% of the patients and the majority of them concerned early-stage disease. Specifically, stage I patients in the new 2023 FIGO staging system decreased in numbers, while stage II increased. This was mainly due to the new IIC substage, which includes all aggressive histological subtypes with any myometrial invasion, so many previously 2009 staged IA and IB 2009 were upshifted. These patients also demonstrated a worse 5-year PFS and 5-year OS compared to patients in stage IA and IB (PFS: 60.2 vs. 86.7 and 76.2 and OS: 75.4 vs. 88.9 and 81). In advanced-stage disease, most of the cases were upshifts from the 2009 IVB stage to the new 2023 IVC stage. However, due to the low number of patients in these substages, no robust statistical analysis was possible. Moreover, the prognostic precision of the 2023 FIGO staging system was tested with several statistical test (AIC, BIC, C-index, likelihood-test), and no difference was found between the two staging systems.

To our knowledge, there are only a few studies in the literature that have compared the two FIGO staging systems. The first study [17] was from three large ESGO-accredited centers in Europe, which included 519 patients, and the majority of them underwent molecular testing. Their results are in accordance with ours, since most of the stage shifts concerned early-stage disease and specifically upshifts to the new substage IIC. However, our results differ in the 5-year PFS and 5-year OS of the new 2023 FIGO staging system and its prognostic impact, because the authors found a superior prognostic precision form the old 2009 FIGO staging system. However, in the survival analyses, only 232 patients were included from two of the three centers, in order to achieve an acceptable long enough follow-up period. Furthermore, the second study [18] was a single-center small retrospective cohort of 161 patients from Korea, where all patients underwent molecular classification. However, the authors stated that POLE mutation was not tested with NGS, which is the way proposed in the ESGO/ESTRO/ESP guidelines to avoid missing any hotspots. The results were similar to ours concerning the stage shifts in early-stage disease.

Last but not least, the latest study [19] was from the USA and included over 100,000 patients with endometrial cancer from the National Cancer Database (NCB). Their results were in accordance with ours for early-stage disease stage shifts, and they also provided data about advanced-stage disease. However, these results, as also stated by the authors, should be interpreted with caution, because none of the patients included underwent molecular testing, and there were no accurate data about LVSI, which is a key element of the new 2023 FIGO staging system.

Our study was conducted in a university, tertiary ESGO-certified center. All the required parameters were collected from an online system, therefore minimizing the percentage of missing important data, and all stage shifts were double checked from the two leading authors. Furthermore, our study has the longest median follow-up period and the highest population in the final analysis. In contrast, the main limitation of our study is the low number of patients with molecular testing and its retrospective nature. Many experts have expressed their reservations about the new 2023 FIGO staging system [20] and especially about the incorporation of the molecular classification. This leads to bias towards recourse-rich centers and/or countries, especially for the *POLE* mutational test, due to its cost and high variability in the testing method that was used. The main point for accelerating the introduction of molecular testing in everyday clinical practice is first to determine which test is the best and most accurate and then lower the cost, in order to make it applicable worldwide.

Future large and carefully designed studies are needed in order to fully understand the implications of the molecular classification in the new 2023 FIGO staging system.

## 5. Conclusions

The new 2023 FIGO staging system better distinguishes early-stage endometrial cancer into its prognostic groups and seems to be as precise as the old 2009 FIGO staging system. Patients should be encouraged to undergo molecular testing.

## Figures and Tables

**Table 1:** Description of 2009 FIGO staging system.

Stage	Description
Stage I	Tumor confined to the corpus uteri
IA	No or less than half myometrial invasion
IB	Invasion equal to or more than half of the myometrium
Stage II	Tumor invades cervical stroma but does not extend beyond the uterus
Stage III	Local and/or regional spread of the tumor
IIIA	Tumor invades the serosa of the corpus uteri and/or adnexa
IIIB	Vaginal and/or parametrial involvement
IIIC	Metastases to pelvic and/or para-aortic lymph nodes
IIIC_1_	Positive pelvic nodes
IIIC_2_	Positive para-aortic lymph nodes with or without positive pelvic lymph nodes
Stage IV	Tumor invades bladder and/or bowel mucosa, and/or distant metastases
IVA	Tumor invasion of bladder and/or bowel mucosa
IVB	Distant metastases, including intra-abdominal metastases and/or inguinal lymph nodes

**Table 2:** Description of 2023 FIGO staging system.

Stage	Description
Stage I	Confined to the uterine corpus and ovary
IA	Disease limited to the endometrium or non-aggressive histological type
IAm_POLEmut_	*POLEmut* endometrial carcinoma, confined to the uterine corpus or with cervical extension, regardless of LVSI or histological type
IA1	Non-aggressive histological type limited to an endometrial polyp or confined to the endometrium
IA2	Non-aggressive histological types involving less than half of the myometrium with no or focal LVSI
IA3	Low-grade endometrioid carcinomas limited to the uterus and ovary
IB	Non-aggressive histological types with invasion of half or more of the myometrium and with no or focal LVSI
IC	Aggressive histological types limited to a polyp or confined to the endometrium
Stage II	Invasion of cervical stroma without extrauterine extension or with substantial LVSI or aggressive histological types with myometrial invasion
IIA	Invasion of the cervical stroma of non-aggressive histological types
IIB	Substantial LVSI of non-aggressive histological types
IIC	Aggressive histological types with any myometrial involvement
IICm_p53abn_	p53abn endometrial carcinoma confined to the uterine corpus with any myometrial invasion, with or without cervical invasion, and regardless of the degree of LVSI or histological type
Stage III	Local and/or regional spread of the tumor of any histological subtype
IIIA	Invasion of uterine serosa, adnexa, or both by direct extension or metastasis
IIIA1	Spread to ovary or fallopian tube (except when meeting stage IA3 criteria)
IIIA2	Involvement of uterine subserosa or spread through the uterine serosa
IIIB	Metastasis or direct spread to the vagina and/or to the parametria or pelvic peritoneum
IIIB1	Metastasis or direct spread to the vagina and/or the parametria
IIIB2	Metastasis to the pelvic peritoneum
IIIC	Metastasis to the pelvic or para-aortic lymph nodes or both
IIIC1	Metastasis to the pelvic lymph nodes
IIIC1i	Micrometastasis
IIIC1ii	Macrometastasis
IIIC2	Metastasis to para-aortic lymph nodes up to the renal vessels, with or without metastasis to the pelvic lymph nodes
IIIC2i	Micrometastasis
IIIC2ii	Macrometastasis
Stage IV	Spread to the bladder mucosa and/or intestinal mucosa and/or distance metastasis
IVA	Invasion of the bladder mucosa and/or the intestinal/bowel mucosa
IVB	Abdominal peritoneal metastasis beyond the pelvis
IVC	Distant metastasis, including metastasis to any extra- or intra-abdominal lymph nodes above the renal vessels, lungs, liver, brain, or bone

**Table 3:** Patients’ characteristics.

		Number of Patients (N)	Percentage (%)
Age (years)		mean: 63.5	SD: 11.6
BMI (kg/m^2^)		median: 32.8	IQR: 28–38.2
BMI categories		median: 2	IQR: 1–4
	<18.5	0	53
	≥18.5–24.9	34	12.9
	≥25–29.9	59	13.7
	≥30–34.4	155	36.6
	≥35	55	12.8
	Missing	168	39
CCI		median: 3	IQR: 2–4
	0–2	115	26.7
	3–4	179	41.5
	≥5	40	9.3
	Missing	97	22.5
Histology			
	Carcinosarcoma	14	3.2
	Clear cell	6	1.4
	Endometrioid	355	82.4
	Mixed	18	4.2
	Mucinous	2	0.5
	Serous	31	7.2
	Undifferentiated	5	1.2
Grade			
	Low	306	71
	High	125	29
LVSI			
	No	343	79.6
	Focal	43	10
	Substantial	45	10.4
Molecular subtypes			
	MMRd	7	1.6
	POLEmut	11	2.6
	p53abn	13	3
	NSMP	2	0.5
	Unknown	398	92.3

**Table 4:** Stage shifts between 2009 and 2023 FIGO staging systems.

		2009 FIGO								
		IA	IB	II	IIIA	IIIB	IIIC1	IIIC2	IVA	IVB
2023 FIGO		n = 195	n = 95	n = 51	n = 10	n = 14	n = 24	n = 20	n = 5	n = 17
IAm_polemut_	n = 2	1	1 (↓)	-	-	-	-	-	-	-
IA1	n = 33	33	-	-	-	-	-	-	-	-
IA2	n = 134	134	-	-	-	-	-	-	-	-
IA3	n = 1	-	-	-	1 (↓)	-	-	-	-	-
IB	n = 63	-	63	-	-	-	-	-	-	-
IC	n = 10	10 (↑)	-	-	-	-	-	-	-	-
IIA	n = 33	-	-	33	-	-	-	-	-	-
IIB	n = 4	1 (↑)	2 (↑)	1	-	-	-	-	-	-
IIC	n = 59	16 (↑)	27 (↑)	16	-	-	-	-	-	-
IICm_p53abn_	n = 3	-	2 (↑)	1	-	-	-	-	-	-
IIIA1	n = 4	-	-	-	4	-	-	-	-	-
IIIA2	n = 4	-	-	-	4	-	-	-	-	-
IIIB1	n = 12	-	-	-	-	12	-	-	-	-
IIIB2	n = 3	-	-	-	1 (↑)	2	-	-	-	-
IIIC1i	n = 11	-	-	-	-	-	11	-	-	-
IIIC1ii	n = 13	-	-	-	-	-	13	-	-	-
IIIC2i	n = 3	-	-	-	-	-	-	3	-	-
IIIC2ii	n = 17	-	-	-	-	-	-	17	-	-
IVA	n = 5	-	-	-	-	-	-	-	5	-
IVB	n = 11	-	-	-	-	-	-	-	-	11
IVC	n = 6	-	-	-	-	-	-	-	-	6 (↑)

**Table 5:** Five-year PFS and OS for the 2009 and 2023 FIGO staging systems.

	2009 Stage			2023 Stage		
	Patients n (%)	5-Year PFS in % (95% CI)	5-Year OS in % (95% CI)	Patients n (%)	5-Year PFS in % (95% CI)	5-Year OS in % (95% CI)
**I**	**290 (67.3)**	**83.3 (78.4, 88.4)**	**86 (81.2, 91.2)**	**243 (56.4)**	**84 (78.9, 89.5)**	**86.6 (81.5, 92.1)**
IA	195 (45.2)	87 (81.8, 92.5)	88.7 (83.4, 94.3)	150 (34.8)	86.7 (81.2, 92.7)	88.9 (83.3, 94.9)
IAm_polemut_				2 (0.5)	n.e.	n.e.
IA1				23 (5.3)	n.e.	n.e.
IA2				124 (28.8)	85.4 (79., 92.4)	88.7 (82.5, 95.3)
IA3				1 (0.3)	n.e.	n.e.
IB	95 (22.)	74.7 (64.8, 86.2)	80.2 (70.5, 91.3)	63 (14.6)	76.2 (64.7, 89.8)	81 (69.7, 94.1)
IC				10 (2.3)	n.e.	n.e.
**II**	**51 (11.8)**	**57.2 (43.8, 74.6)**	**62.8 (48.9, 80.7)**	**99 (23.0)**	**66.9 (56.7, 79.0)**	**70.2 (59.6, 82.8)**
IIA				33 (7.7)	61 (45.3, 82.2)	62.5 (46.5, 84.0)
IIB				4 (0.9)	n.e.	n.e.
IIC				59 (13.7)	60.2 (45.4, 79.8)	75.4 (61.9, 91.8)
IICm_p53abn_				3 (0.7)	n.e.	n.e.
**III**	**68 (15.8)**	**49.4 (36.4, 67.1)**	**48.0 (34.8, 66.1)**	**67 (15.5)**	**49.3 (36.3, 67.0)**	**47.9 (34.8, 66.0)**
IIIA	10 (2.3)	n.e.	n.e.	8 (1.9)	n.e.	n.e.
IIIA1				4 (0.9)	n.e.	n.e.
IIIA2				4 (0.9)	n.e.	n.e.
IIIB	14 (3.2)	46.3 (21.3, 100)	46.3 (21.3, 100)	15 (3.5)	46.8 (21.6, 100)	46.8 (21.6, 100.0)
IIIB1				12 (2.8)	19.3 (23.0, 100)	49.5 (19.3, 23.1)
IIIB2				3 (0.7)	n.e.	n.e.
IIIC				44 (10.2)	52.4 (37.3, 73.8)	51.9 (36.4, 73.9)
IIIC1	24 (5.6)	48.3 (29.5, 79)	51.2 (32.2, 81.3)	24 (4.9)	48.3 (29.5, 79)	51.2 (32.2, 81.3)
IIIC1i				11 (2.6)	60.6 (18.4, 33.4)	37.9 (8.9, 100)
IIIC1ii				13 (3.0)	55 (33.4, 90.6)	33.2 (13.6, 81)
IIIC2	20 (2.6)	57.3 (35.7, 92.1)	52.4 (30.0, 91.5)	20 (4.6)	52.4 (30.0, 91.5)	52.4 (30.0, 96.5)
IIIC2i				3 (0.7)	n.e.	
IIIC2ii				17 (3.9)	55 (33.4, 90.6)	51.1 (28.9, 90.3)
**IV**	**22 (5.1)**	**n.e.**	**n.e.**	**22 (5.1)**	**n.e.**	**n.e**
IVA	5 (1.2)	n.e.	n.e.	5 (1.2)	n.e.	n.e.
IVB	17 (3.9)	n.e.	n.e.	11 (2.6)	n.e.	n.e.
IVC				6 (1.4)	n.e.	n.e.

**Table 6:** Histopathological characteristics of upshifted patients.

		Number of Patients (N)	Percentage (%)
Histology			
	Carcinosarcoma	3	6.2
	Clear cell	2	4.2
	Endometrioid	24	50
	Mixed	10	20.8
	Mucinous	-	-
	Serous	8	16.7
	Undifferentiated	1	2.1
Grade			
	Low	7	14.6
	High	41	85.4
LVSI			
	No	30	62.5
	Focal	13	27.1
	Substantial	5	10.4

## Data Availability

In accordance with the journal’s guidelines, the data presented in this study are available on request from the corresponding author for the reproducibility of this study.
